# Editorial: Circulating tumor DNA in cancer: a role as a response and monitoring “next-generation” biomarker in cancer therapy

**DOI:** 10.3389/fonc.2023.1210866

**Published:** 2023-06-01

**Authors:** Saeid Latifi-Navid, Reza Safaralizadeh, Lixuan Wei

**Affiliations:** ^1^ Department of Biology, Faculty of Sciences, University of Mohaghegh Ardabili, Ardabil, Iran; ^2^ Department of Biology, Faculty of Natural Sciences, University of Tabriz, Tabriz, Iran; ^3^ Department of Molecular Pharmacology and Experimental Therapeutics, Mayo Clinic, Rochester, MN, United States

**Keywords:** ctDNA, cancer therapy, response, monitoring, biomarker

In recent years, circulating tumor DNA (ctDNA) has gained substantial promise as a sensitive biomarker for tumor diagnosis, prognosis and response monitoring of a wide range of treatment modalities. This sensitive biomarker has been shown to be effective in detecting residual disease and diagnosing recurrence, and in tumor-specific adjuvant therapy and targeted therapy (1), Peng et al. A ctDNA biomarker is also innately sensitive and specific for metastatic cancer (2, 3). In this way, ctDNA as a liquid biopsy may represent an exciting era in cancer management, but there remain some challenges. Specifically, we need to 1) learn more about ctDNA’s biological characteristics (such as its size, existing form, and mechanism of release), 2) improve the sensitivity of the method for detecting ctDNA, and 3) validate its translation into routine clinical practice through a variety of clinical trials and multi-center cohorts. This Research Topic embodies 10 multidisciplinary manuscripts (original research and critical reviews) focused on multifaceted aspects related to “CtDNA in Cancer”.

It is essential to understand ctDNA biology in order to develop techniques that allow its analysis. As a result, Sanchez-Herrero et al. offered an overview of ctDNA biological features, including size and structure, mechanisms of shedding and clearance, and physiopathological factors that influence ctDNA levels. Moreover, Peng et al. discussed the clinical applications and challenges of ctDNA and minimum residual disease (MRD) in solid tumors. An MRD test helps to evaluate the patient’s prognosis, treatment response, and recurrence risk. They discussed how ctDNA can be used to monitor MRD in solid tumors, such as breast cancer, lung cancer, and colon cancer. Overall, ctDNA-based MRD detection can improve patient outcomes in cancers and assist in clinical decision-making. In a review article, Lam et al. examined ctDNA as a biomarker for gastro-esophageal, colorectal (CRC), and pancreaticobiliary cancers. They discussed how this biomarker’s unique strengths might be used in improving management of gastrointestinal cancers. During palliative care, ctDNA monitoring can be used to detect and track clonal variants linked to acquired resistance to immune-checkpoint inhibitors and targeted therapies. Moreover, ctDNA may be used to guide therapeutic re-challenge for patients who have taken targeted therapies in the past.

Diefenbach et al. developed an NGS panel to identify melanoma ctDNA that includes 15 top gene mutations including the *TERT* promoter. They analyzed 21 melanoma samples from stage III or IV patients who were either untreated or receiving therapy for their disease. The custom panel detected 14/21 (67%) patients with mutations in *BRAF/NRAS/TERT* promoter, one of whom contained a *TERT* C250T mutation in one negative sample for *BRAF* and *NRAS* mutation. They plan to expand their custom panel to 50 genes in order to improve detection rates of stage IV melanoma to >90%. Liquid biopsy approaches based on ctDNA may be an effective method of interrogating gastrointestinal stromal tumors (GISTs). Ko et al. tested plasma samples from 46 patients with a customized 29-gene Archer^®^ LiquidPlex™ target panel. This is an attractive non-invasive method for obtaining relevant clinical data during disease progression.

Endocrine therapy is a cornerstone of therapy for hormone receptor-positive (HR+), HER2-negative metastatic breast cancer (mBC). Urso et al. evaluated the concordance between ctDNA and *ESR1* status in metastatic tumors. A 91% concordance rate was found between tumor tissue and plasma *ESR1* status. The study showed that liquid biopsy could be an alternative to tissue biopsy for the assessment of *ESR1* mutations in mBC. By sequencing the entire exome of cfDNA, Lee et al. identified novel genetic mutations linked to drug resistance in lung cancer and CRC patients treated with EGFR-targeted therapies and chemotherapy. Sixteen genes in CRC and seven genes in lung cancer were found. Additionally, TTN R7415H and ADAMTS20 S1597P mutations in CRC, as well as the GPR155 I357S mutation in lung cancer, were frequently detected during acquired resistance. This indicates that these mutations play a critical role in acquired resistance to chemotherapy. It is estimated that 3~5% of non-small cell lung cancers (NSCLCs) have leptomeningeal metastases (LM). As indicated by Bai et al., CSF ctDNA from a lung adenocarcinoma patient showed oncogenic mutations before CSF cytology and MRI confirmed LM, indicating CSF ctDNA as a potential early detection tool. A study by Wu et al. examined ctDNA mutated genes, prognosis, and the association between the altered genes in ctDNA and clinical parameters in lymphoma. They proposed that NGS-based analysis of ctDNA mutations can reveal heterogeneities in lymphoma subtypes, which could offer new therapeutic targets, insights into genomic evolution, and new approaches to risk-adaptive therapies.

Interestingly, Chan et al. compared tumor-informed versus tumor-agnostic approaches to ctDNA analyses in CRC patients. The benefits of a single-time point ctDNA analysis were compared with serial monitoring of ctDNA after definitive treatment. They concluded that longitudinal monitoring of tumor-informed ctDNA is highly analytically sensitive, with a low probability of false-positive rate due to clonal hematopoiesis mutations, as well as improved sensitivity to detect recurrence, which may modify CRC clinical management.

Altogether, the original articles and reviews collected in this Research Topic provide original insights and critical perspectives for translating ctDNA into clinical practice and management of patients suffering from malignant tumors.

## Author contributions

The work has been approved for publication by all authors who have made substantial, direct, and intellectual contributions.
